# Interaction of the AKT and β-catenin signalling pathways and the influence of photobiomodulation on cellular signalling proteins in diabetic wound healing

**DOI:** 10.1186/s12929-023-00974-8

**Published:** 2023-09-21

**Authors:** Sandy Winfield Jere, Heidi Abrahamse, Nicolette Nadene Houreld

**Affiliations:** Laser Research Centre, Faculty of Health Sciences, University of Johannesburg, P.O. Box 17011, Doornfontein, 2028 South Africa

**Keywords:** Cell survival, Cellular signalling, GSK3β, PI3K/AKT, Wnt/β-catenin, Diabetes, Diabetic wound

## Abstract

The induction of a cells destiny is a tightly controlled process that is regulated through communication between the matrix and cell signalling proteins. Cell signalling activates distinctive subsections of target genes, and different signalling pathways may be used repeatedly in different settings. A range of different signalling pathways are activated during the wound healing process, and dysregulated cellular signalling may lead to reduced cell function and the development of chronic wounds. Diabetic wounds are chronic and are characterised by the inability of skin cells to act in response to reparative inducements. Serine/threonine kinase, protein kinase B or AKT (PKB/AKT), is a central connection in cell signalling induced by growth factors, cytokines and other cellular inducements, and is one of the critical pathways that regulate cellular proliferation, survival, and quiescence. AKT interacts with a variety of other pathway proteins including glycogen synthase kinase 3 beta (GSK3β) and β-catenin. Novel methodologies based on comprehensive knowledge of activated signalling pathways and their interaction during normal or chronic wound healing can facilitate quicker and efficient diabetic wound healing. In this review, we focus on interaction of the AKT and β-catenin signalling pathways and the influence of photobiomodulation on cellular signalling proteins in diabetic wound healing.

## Background

Protein components that serve as cellular receptors, cytoplasmic couriers, and nuclear effectors constitute the cellular signalling pathway for signal transduction and gene expression. Cell receptor proteins are transmembrane, having an extracellular domain to which growth factors (GFs)/cytokines bind, and an intracellular enzyme linked domain. To induce and convey signals to the cytoplasmic couriers and nuclear effectors, cell receptor proteins interact with distinctive chemical messengers including ligands such as GF and cytokines. Ligands are released by several cell types to signal themselves or others. Ligand binding creates an intermediate form of the receptors cytosolic domain, and this change instigates a sequence of downstream biochemical reactions [1–3]. Receptors are classified into five groups: cell surface, enzyme-linked, internal, G-protein-coupled (GPC), and ion channel. When the released ligand binds to receptors on the same cell that released them, the altered cellular behavior is said to be autocrine. Ligands can also be released and passed directly across to neighboring cells to form gap junction cell activation, and when they are passed onto nearby cells, or travel through the bloodstream to distant cellular receptors, it is referred to as paracrine and endocrine cell activation, respectively [4, 5].

Cellular signalling is mediated by molecular alterations that switch on and off a given cellular response or function, and this is accomplished via several mechanisms including desensitising receptors to their ligands, degradation of signalling proteins, and restoration of normal concentration of the ligands [6]. Moreover, the presence of specific extracellular and transmembrane signalling competitors and activators influence cellular reaction. For instance, the Wnt/β-catenin signalling pathway is inhibited by extracellular factors including frizzled-related proteins (FRPs), cerberus, dickkopf proteins (Dkks), sclerostin domain-containing 1 (Wise/SOST), Wnt-inhibitory factor 1 (WIF-1), insulin-like GF binding protein 4 (IGFBP-4), and transmembrane factors including Wnt-activated inhibitory factor 1 (Waif1/5T4), and adenomatosis polyposis coli down-regulated 1 (APCD1). Literature shows that Norrin and R-spondin advances the Wnt/β-catenin pathway by increasing stabilisation and phosphorylation of β-catenin [7]. The complexity and specificity of cellular signalling can also be controlled by compartmentalised signalling from distinct centers such as organelle specific pools of mitogen-activated protein kinases (MAPKs) within the golgi apparatus that can rapidly switch a particular signalling pathway on or off [8]. Defects in cellular signalling or pathogenic distress can cause numerous cellular alterations through the modification of signalosomes leading to the pathways hyper- or hypo-sensitive condition, and continuous and uncontrolled signalling can be detrimental [9]. Several factors including genetic and epigenetic changes can cause uncontrollable activation of cellular signalling pathways, resulting in disadvantageous cellular outcomes including over proliferation and attenuated getaway mechanisms that under normal circumstances control their growth, migration, and survival. These alterations can lead to the development of cancer and its progression, and have also been shown to be involved in delayed healing in chronic diabetic wounds [10].

Diabetes mellitus (DM) is a chronic disease that arises when the pancreas is not able to produce insulin or when cells do not make good use of insulin, a phenomenon that leads to elevated blood glucose levels (hyperglycaemia). In 2021, an estimated 537 million adults aged between 20 and 79 years were living with diabetes, and this number is projected to increase to 783 million by 2045. In the same year, DM was responsible for roughly 6.7 million deaths [11]. The increasing global incidence of diabetes is associated with a high occurrence of chronic diabetic wounds, and this complication is characterised by disrupted molecular factors involved in normal wound healing [12]. According to Abbas and Boulton [13], almost 25% of diabetic patients develop chronic wounds of the foot, with recurrence rates of 70% after treatment, and out of these, 85% may require lower limb amputation. They further pointed out that diabetic patients with one amputated lower limb are at risk of developing a chronic wound on the remaining limb, with a 70% mortality rate within five years after the first amputation.

**Fig. 1:**
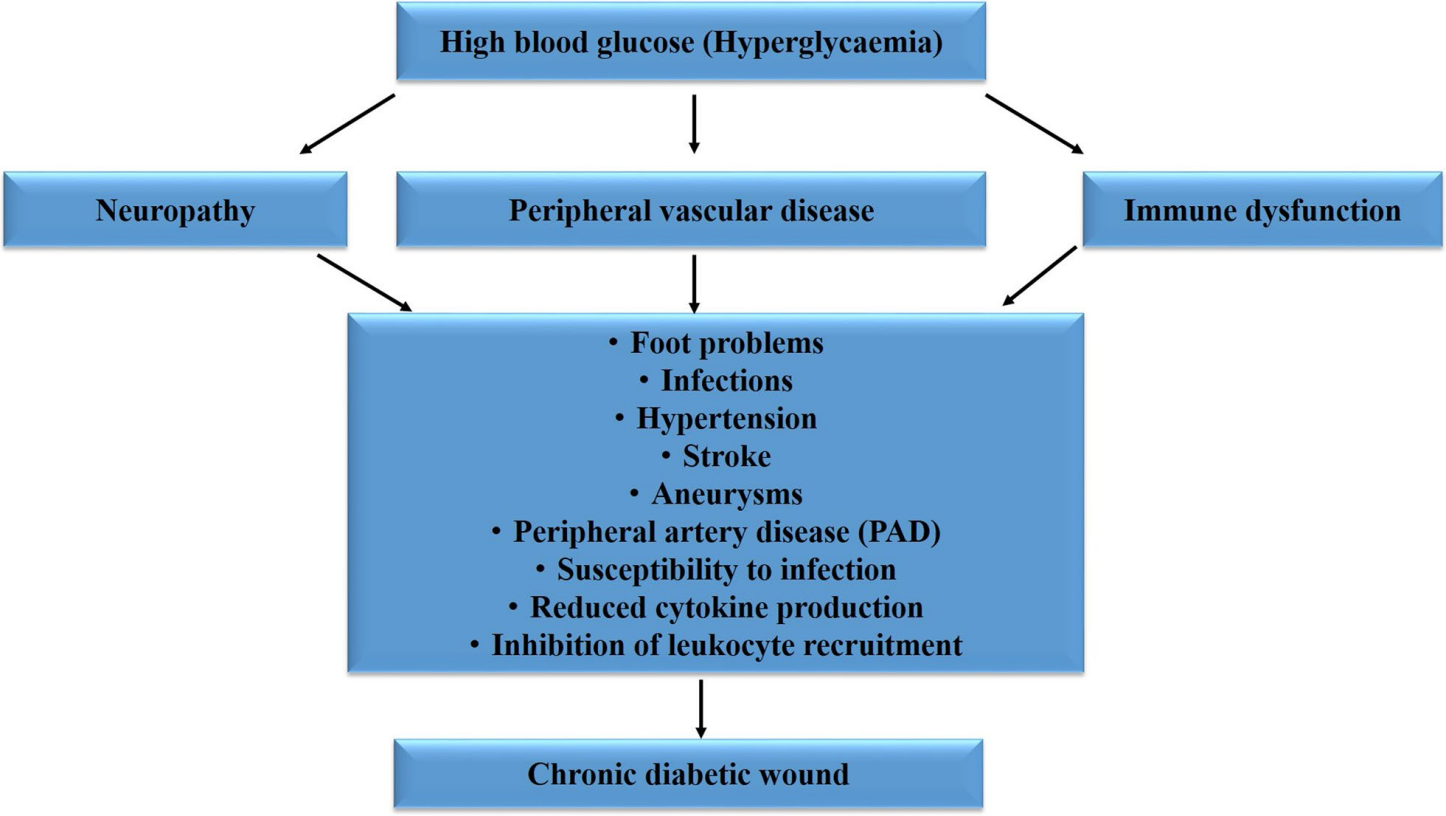
Chronic hyperglycaemia and development of chronic diabetic wounds

Chronic hyperglycaemia is linked to impairment and failure of various organs and tissues (Fig. 1), and is one of the major contributing factors for the development of wound chronicity [14]. When diabetic patients develop chronic wounds, they become at high risk of developing distressing complications such as infection and limb amputation. In DM, altered tissue repair is related to abnormalities including neuropathic, vascular, and immune function. Standard treatment methods that tightly control blood glucose levels and thorough wound care are essential. However, the prognosis for the healing of diabetic wounds is very poor [11]. Typically, uncontrolled DM, oxidative stress, and heightened cell apoptosis leads to the development of chronic non-healing wounds. Hyperglycaemia-induced protein glycation generates superoxide free radicals that lead to the formation of reactive products, lipid peroxidation, and damage to cellular molecules [15]. Diabetic wounds develop due to poor glycaemic control, peripheral vascular disease, underlying damage to the nervous system (neuropathy), and inadequate foot care. In the presence of neuropathy and peripheral vascular disease, the healing of bruises or injuries may be delayed, leading to the development of chronic diabetic wounds. Mostly, diabetic wounds of the foot develop in areas that encounter persistent pressure and trauma, where hardened skin breaks due to repeated pressure in the presence of motor neuropathy [16, 17]. Sensory neuropathy is the main cause for sensory loss to trauma, and the consequence of persistent trauma on a hardened skin is subcutaneous haemorrhage, which eventually erodes and becomes ulcerated [16].

Characteristically, the presence of hyperglycemia, hypertension, and hypercholesterolemia in DM are risk factors for several complications such as damage to the nervous system, renal system (nephropathy), and eyes (retinopathy), and development of cardiovascular and peripheral vascular diseases and stroke. It is suggested that the control of blood pressure, glucose, and cholesterol levels can reduce the risk of developing these complications [18]. The growing number of diabetic patients with chronic wounds has caused serious social and economic burdens worldwide, and routine therapeutic strategies have been challenging for the patient as well as the healthcare system, mostly typified with high costs and unsatisfactory results. In this regard, the development of innovative cost-effective therapeutic methods that can improve patient outcomes is gaining increased attention [19, 20].

### Interaction between AKT and β-catenin pathways in cell survival

**Fig. 2:**
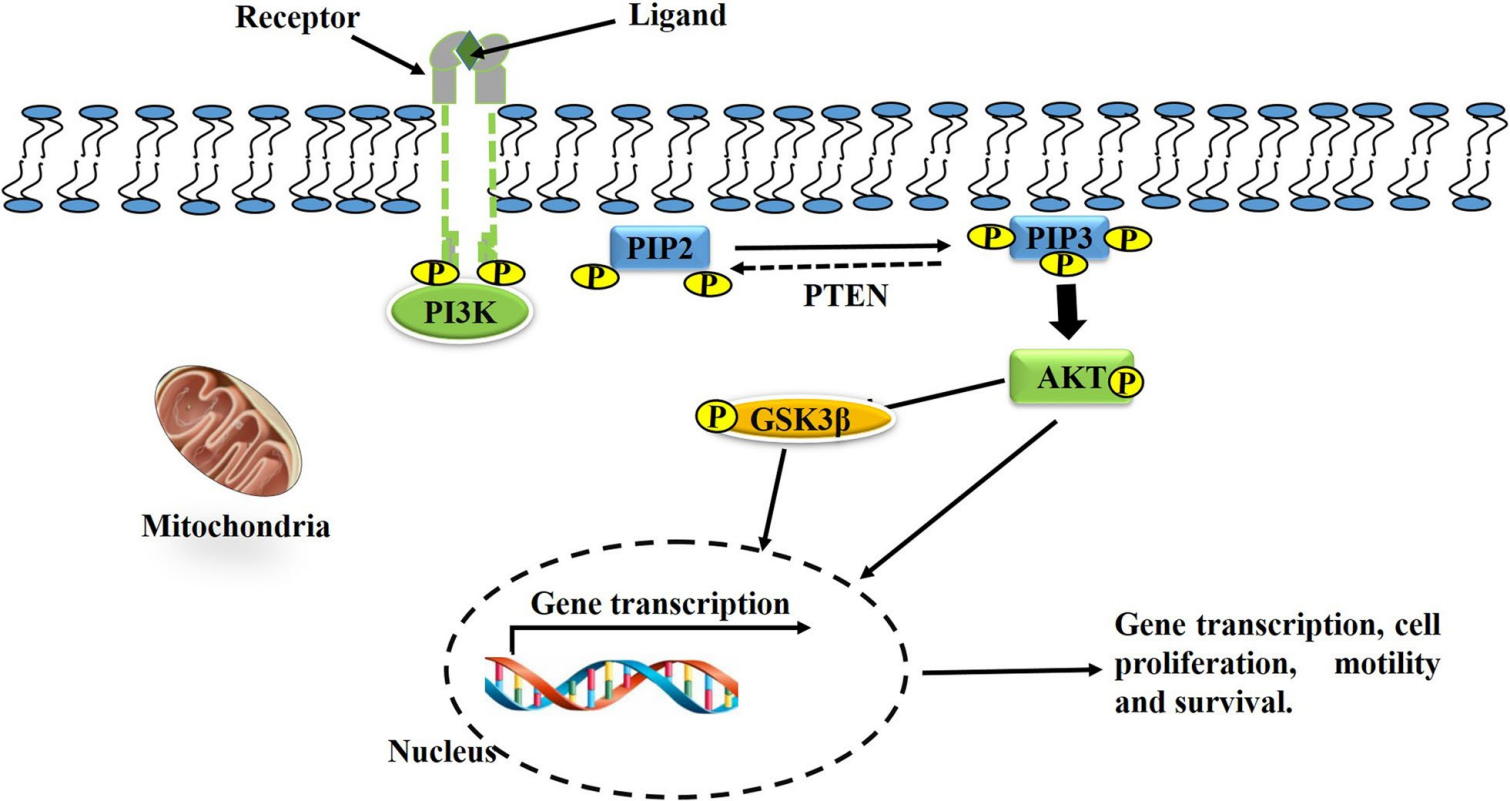
Phosphatidylinositol 3 kinase/Protein Kinase B (PI3K/AKT) signalling

**Fig. 3:**
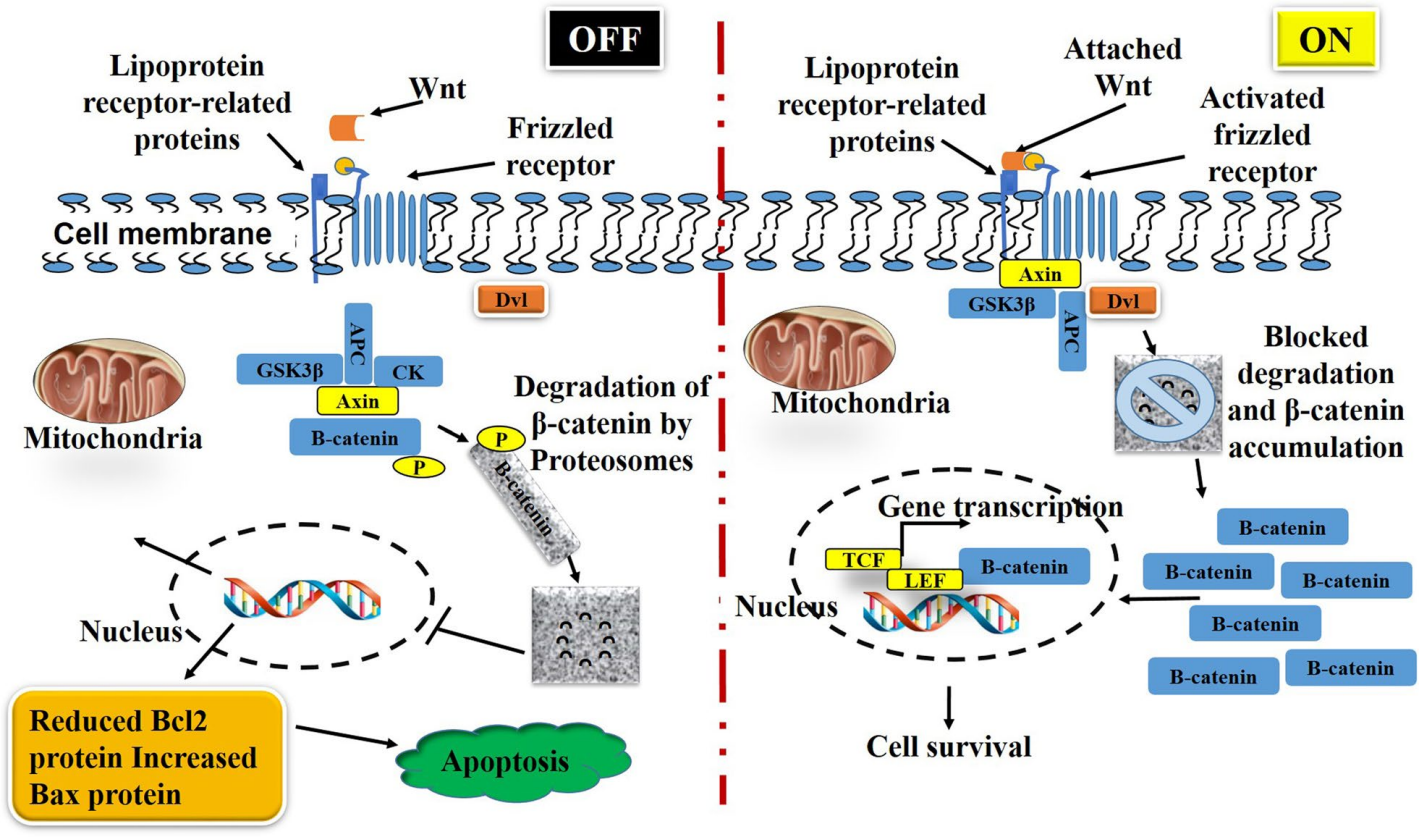
The cellular Wnt/β-catenin pathway

The Phosphoinositide 3-kinase (PI3K)/AKT and wingless/integrated (Wnt)/β-catenin pathways are basic regulators of cell proliferation, differentiation, development, and apoptosis, and their relationship is so close such that they have become a distinctive therapeutic target [21]. AKT plays a critical function in cellular survival, and is activated when ligands bind to cell surface receptor tyrosine kinases (RTKs), resulting in the activation of the PI3K/AKT pathway (Fig. 2). PI3K/AKT signalling is regulated by the tumour suppressor gene phosphatase and tensin homologue (PTEN), representing both lipid and protein phosphatase that negatively regulates PI3K/AKT signalling via the dephosphorylated phosphatidylinositol-3,4,5-trisphosphate (PIP3) to phosphatidylinositol 4,5-bisphosphate (PIP2) [22]. The Wnt/β-catenin genes encode for a group of secreted GF proteins, and in humans 19 Wnt genes have been identified. Wnt proteins play a critical role in regulating cell proliferation, migration, and fate. Alternatively, β-catenin, a 781 amino acid protein, is encoded by catenin beta 1 (CTNNB1), and cytoplasmic β-catenin is continuously degraded by an adenomatous polyposis coli (APC)/GSK3β/Axin destruction complex that switches off the pathway which, in the presence and binding of Wnt, is inhibited, resulting in the Wnt/β-catenin pathway switching on (Fig. 3). When the Wnt/β catenin pathway is switched on and is activated, there is increased levels of cytoplasmic β-catenin that is translocated into the nucleus [23].

Cytosolic build-up of β-catenin promotes the stability of nuclear p53, and reduced expression of caspases, c-myc, and regulation of B-cell lymphoma 2 (BCL-2) and cell apoptosis. In the nucleus, β-catenin acts as a transcriptional co-activator that interacts with T-cell factor/lymphoid enhancer-binding factor (TCF/LEF) to form a transcription regulator for genes related to cell proliferation, survival and cancer [24]. The PI3K/AKT and Wnt/β-catenin pathways are essential in the regulation of all the phases of the wound healing process. A wound showing no signs of healing for more than eight weeks becomes chronic, mostly due to DM and reduced cell signalling for cellular migration, proliferation, and survival [10]. Increased blood glucose concentration affects cellular signalling and inhibits cellular proliferation, migration, and angiogenesis, and ultimately wound healing. According to Zhang et al. [25], high glucose prevents the differentiation of stem cells via the reduced expression of cytosolic β-catenin, epidermal growth factor receptor (EGFR), and cyclin D1. Furthermore, they revealed a close interaction between Wnt/β-catenin and P13K/AKT signalling during inflammatory responses, cell proliferation and differentiation, and wound healing. In agreement to these suggestions, Wei et al. [14], demonstrated the involvement of P13K/AKT signalling proteins in angiogenesis and fibroblast activation during diabetic wound healing, and the PI3K/AKT facilitated inhibition of GSK3β affects the stability and nuclear translocation of β-catenin.

PI3K/AKT and Wnt/β-catenin signalling share receptors, including GSK3β, receptor frizzled (FZD), adaptor dishevelled (Dvl), dishevelled egl-10 and pleckstrin (DEP) domain-containing mTOR interacting protein (DEPTOR), and eukaryotic translation initiation factor 4E (eIF4E) that are engaged in both pathways. GSK3β is inhibited when both PI3K/AKT and Wnt/β-catenin signalling are activated via distinctive upstream activation [26]. Prossomariti and colleagues [27], clarified the detailed interaction of these pathways through the GSK3β FZD, Dvl, DEPTOR and eIF4E receptors. The existence of an inter-connectivity between the PI3K/AKT and Wnt/β-catenin pathways has been observed during cellular proliferation and survival. Recently, Fleming-de-Moraes et al. [28], observed an increased activation of these two pathways and enhanced cell migration and proliferation when both signalling pathways were simultaneously stimulated with IGF 1 and Wnt. However, these effects were reduced in the presence of a specific PI3K inhibitor, suggesting some dependency involving these two signalling pathways [29]. The EGF-induced stimulation of β-catenin is dependent on the activation of AKT, and EGFR activation causes the Wnt-free β-catenin activation. Cross communication between the PI3K/AKT and Wnt/β-catenin pathways is critical, and therapeutic options targeting major proteins for the PI3K/AKT and Wnt/β-catenin pathways may affect cellular function and wound healing [30].

### Molecular defects in chronic diabetic wounds

The development of chronic diabetic wounds is mostly caused by diminished GF production, cellular migration, proliferation, angiogenic response, and collagen accumulation, and the unbalanced accumulation of extracellular matrix (ECM) components and matrix metalloproteinases (MMPs) [31, 32]. In addition, there are suggestions that the reduced activity of various signalling pathways including insulin, AMP-activated protein kinase (AMPK), and ligand-activated transcription factor pathways such as peroxisome proliferator-activated receptors (PPARs), may play a critical role in the development of chronic diabetic wounds [33, 34]. Stojadinovic et al. [35], alluded that delayed diabetic wound healing may be affected by overexpression of c-myc and nuclear localisation of β-catenin coupled with reduced and irregular localisation of EGFR, and initiation of glucocorticoid signalling. Recent reports indicate that variations in the signalling pathways such as the AKT/mammalian target of rapamycin; mTOR, (AKT/mTOR), results in the reduction of diabetic wound healing [36, 37].

**Fig. 4:**
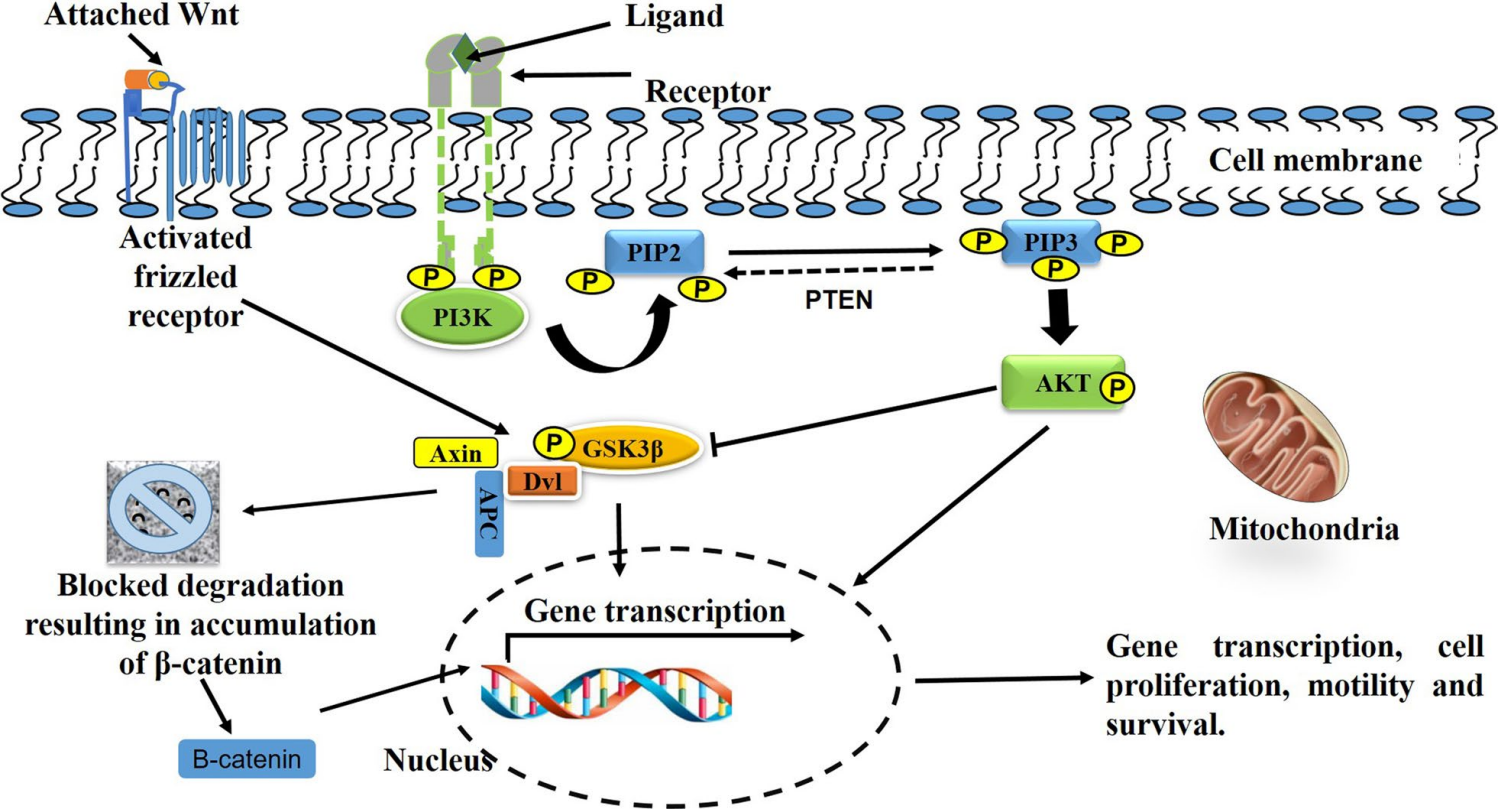
The interaction between Wnt/β-catenin and P13K/AKT signalling

The activation of the insulin pathways, including insulin receptor/Src Homology 2 (IR/SH2), SHC signalling adaptor protein/extracellular signal regulated kinases (SHC/ERKs), and IR/PI3K/AKT generally initiates wound healing [36, 38]. In this regard, Yu et al. [39], reported that topical application of insulin stimulates diabetic wound healing. In their study, insulin treated diabetic wounded rats showed an increase in activated AKT and GSK3β. GSK3β proteins are related to several signalling pathways including Wnt/β-catenin, nuclear factor kappa B (NF-κB), AKT, and nuclear factor erythroid 2 related factor 2 (Nrf2). Dysregulated GSK3β signalling is associated with the development of inflammatory syndromes, diabetic wounds, nephropathy, and retinopathy, and plays a critical role in angiogenesis and modification of the diabetic wound healing process [40]. Although the mechanisms for GSK3β regulation during PI3K/AKT and Wnt/β-catenin signal transduction is partly understood, it is well known that PI3K/AKT inhibits the activity of GSK3β kinase (Fig. 4), one of the key components of the β-catenin degradation and phosphorylation complex. Increased phosphorylation of β-catenin is central to its ubiquitination, degradation and reduced cellular activity [41]. To show the interaction and crosstalk between PI3K/AKT and Wnt/β-catenin pathways, Saraceno et al. [42] demonstrated that perinatal asphyxia (PA) affects the IGF receptor (IGFR)/PI3K/AKT/GSK3β/β-catenin signalling pathways in neuroprotection and neuronal plasticity. In agreement to these observations, Li et al. [43], demonstrated that the activation of human amniotic mesenchymal stem cells (hAMSC) is mediated by PI3K/AKT dependent GSK3β/β-catenin signalling to yield a favorable therapeutic benefit for skin wound healing. When Hoke et al. [44], analysed the expression of signalling protein ratios for PI3K and IGFR, mTOR and IGFR, p53 and IGFR, cyclooxygenase 2 (COX 2) and IGFR, BCL-2-antagonist/killer (BAK) and IGFR, and Caspase 9 and IGFR, they noted a considerable relationship of the values to the healing outcomes in chronic diabetic wounds. In these observations, AKT was seen to be a central regulator for most of the signalling pathways involved in the regulation of cellular migration, proliferation, differentiation, and wound healing [45].

### Management of chronic diabetic wounds

The management of chronic diabetic wounds involves the use of different agents that affect molecular targets by, among others, up- or down-regulating the expression of GFs, MMP, collagen synthesis and degradation, and activation of angiogenesis. GFs are critical for tissue restoration, and due to their diversity in action they have become an attractive treatment agent for stimulating tissue restoration in chronic diabetic wounds. GFs facilitate the movement of cells into the wound, and increase their proliferation and deposition of ECM [46, 47]. Typically, the cellular spatial and temporary release of GFs in response to injury is to make best use of regenerative benefits and reduce side effects to hasten wound healing [12]. There are several families of GFs that are expressed by different cell types involved with wound healing including, but not limited to, epidermal growth factor (EGF), vascular-endothelial growth factor (VEGF), fibroblast growth factor (FGF), platelet-derived growth factor (PDGF), Wnt, and transforming growth factor (TGF). They stimulate a cascade of cellular signalling pathways, including the PI3K/AKT and β-catenin pathways for different cellular functions that advance wound healing [48, 49]. However, MMP mediated ECM and GF/cytokine mortification in chronic diabetic wounds reduce their bioavailability for effective wound healing. Hence, techniques that may deliver stimulators and inhibitors of GFs, angiogenesis, MMP, ECM deposition, gene transcription, stem cell infiltration, and cell rejuvenation in diabetic wound healing are desirable [48, 50].

EGFR promotes the proliferation and movement of cells including keratinocytes and fibroblasts, improves collagen production and ECM synthesis, and its role in normal and uncontrolled wound healing has been expansively investigated [51]. The activation of EGFR has been identified as a mechanism through which GFs control normal and uncontrolled cellular function, and this has led to the development of EGFR inhibitors for cancer treatment and topical GF application to enhance cutaneous wound healing. Therapies targeting EGF/EGFR have shown to be beneficial for the management of both cancer and cutaneous wounds [52]. Wei et al. [53], elucidated that recombinant human EGF (rhEGF) promote epithelial and fibroblast cell proliferation and migration in vitro, and that vacuum sealing drainage (VSD) in combination with rhEGF effectively stimulate wound healing compared to other treatment methods in vivo. However, the chronic wound environment and increased MMPs readily degrades exogenous GFs [54, 55]. The cellular release of VEGF in response to tissue injury promotes angiogenesis and the healing of chronic diabetic wounds [56]. The release of VEGF is induced in the presence of hypoxia, and advances reepithelialisation and increased blood vessel formation during the wound healing process. The use of VEGF treatments is inhibited mainly due to vasopermeability, and in clinical trials, recombinant VEGF has shown minor clinical benefits requiring further studies on their effect in chronic wound healing. Several synthetically derived GFs for the treatment of chronic wounds are available, and comes in many forms including injections, gels, solutions, ointments, and creams. However, the efficacy of these treatment techniques for chronic diabetic wounds is not satisfactory [57, 58]. Usually, the treatment of diabetic wounds involves different techniques and agents that affect molecular targets. For example, dressings ranging from silk, cotton, or linen gauze pads and sponges, to transparent absorbent acrylic pads, hydrocolloids and hydrofibers, or topical cadexomer iodine, honey, hypochlorous acid, sterile saline, and superoxidised therapeutic techniques among others, have been developed [59]. However, due to short term benefits, and inadequate evidence of efficacy and cost-effectiveness of the available treatment methods for chronic diabetic wounds, several other novel treatment methods including photobiomodulation (PBM), formally known as low level light/laser therapy (LLLT), have been suggested [60, 61].

### Photobiomodulation therapy (PBMT) in chronic diabetic wounds

**Fig. 5:**
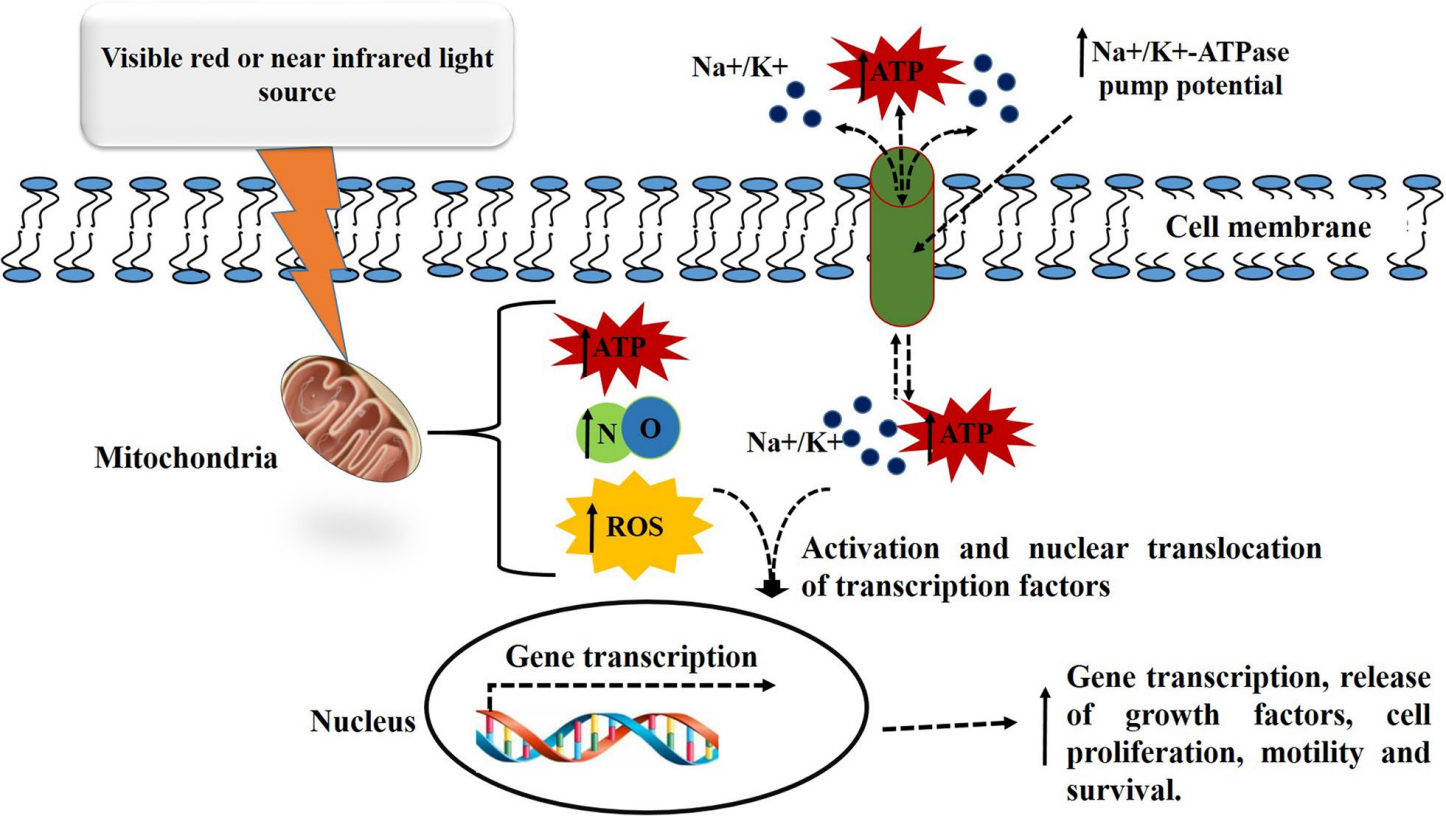
Mechanism of photobiomodulation (PBM) therapy

Photobiomodulation therapy (PBMT) is the use of red and near infra-red light over wounds, lesions, or injuries to improve tissue and wound healing, and lessen inflammation and pain. It has been defined as an effective and low-priced commercially obtainable treatment method that can easily be used at home or in hospitals with an excellent safety profile [62, 63]. PBM was first pioneered in 1983 by Endre Mester, and involves nonthermal and low powered noninvasive lasers or light emitting diodes (LEDs). At a cellular level, PBM works by influencing different biological processes involved in tissue repair, regeneration, and wound healing. It has a biphasic dose reaction relationship, promoting wound healing at lower doses, while harmful or no effect is observed at elevated levels. It is an effectual treatment method used as an adjuvant for both acute and chronic wounds, skin restoration, musculoskeletal disorders, pain management and hair growth [64]. PBM increases the cell membrane potential and the performance of the sodium–potassium pump (Na + /K±ATPase), resulting in increased adenosine triphosphate (ATP) production (Fig. 5). The resultant increase in ATP is useful for the normalisation of cell activity to promote tissue restoration [65].

**Fig. 6:**
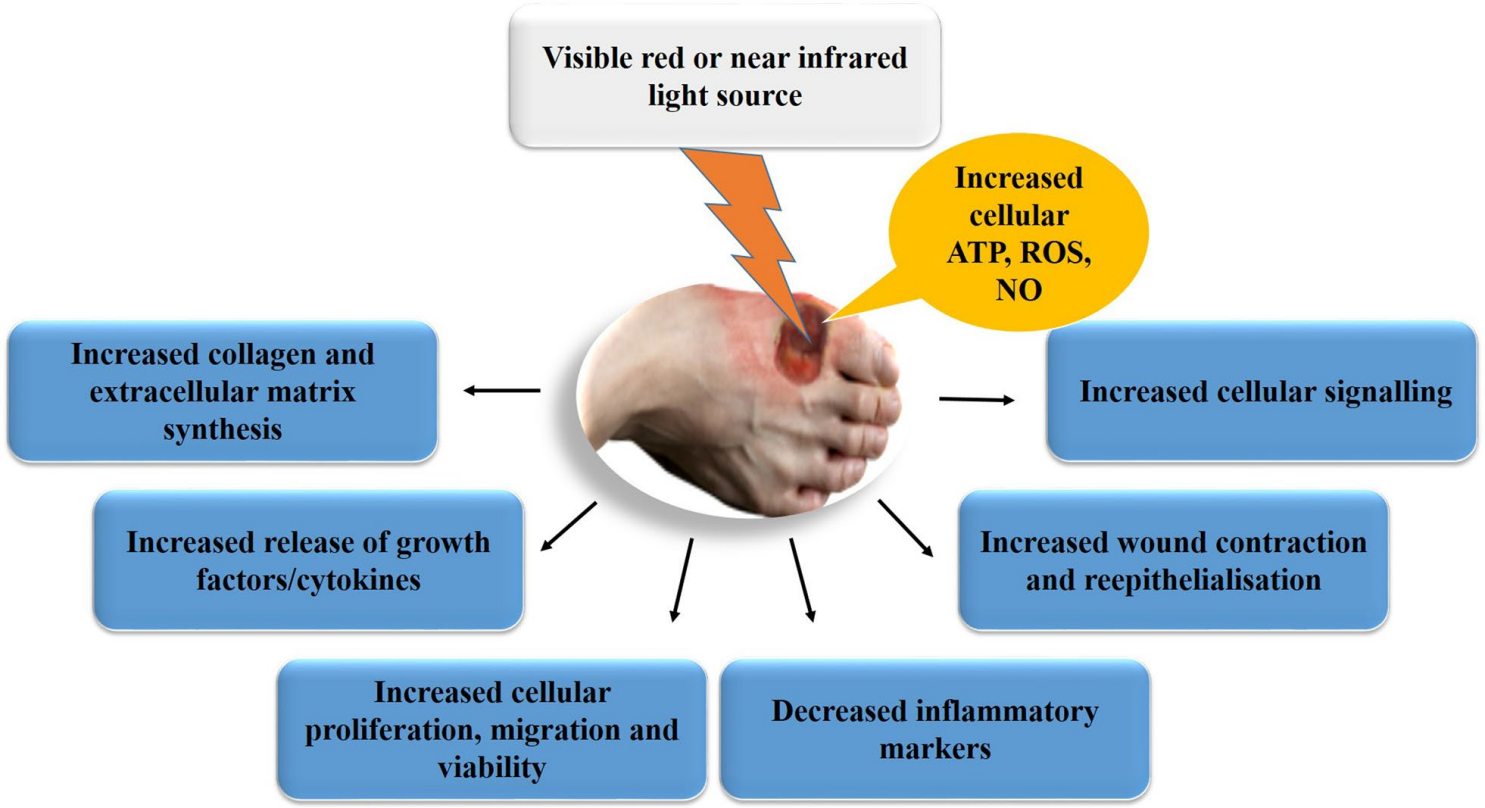
The therapeutic mechanism of photobiomodulation (PBM) in chronic diabetic wounds

PBM is frequently used at wavelengths ranging between 600 and 1100 nm and fluences between 1 and 4J/cm^2^ with power output between 10 and 90 mW [64–66]. Literature suggests that PBM regulates the effects of high blood glucose on endothelial cells, reduces the concentration of TNF-α, augments proliferation of fibroblast cells, and quickens tissue repair in diabetic chronic wounds [67]. Lenifa et al. [68] and Santos et al. [69], reported accelerated wound healing in PBM treated chronic wounds, mainly via the release of cytokines and GFs, interleukin 1 alpha (IL-1α) and interleukin 8 (IL-8), fibroblast cell proliferation, migration and differentiation, and wound restoration (Fig. 6). Furthermore, PBM has been demonstrated to increase the expression of PDGF and TGF beta (TGF-β), and escalate the production of collagen and ECM to promote wound healing [70]. A photochemical response to PBM starts when the photon energy is absorbed by photoreceptors, including cytochrome c oxidase (CCO) on the electron transport chain, resulting in the increased production of ATP, reactive oxygen species (ROS), and nitric oxide (NO) in the cell [71]. Khan et al. [72], suggested that the PBM mediated activation of endogenous TGF-β1 plays a vital role in wound healing. During the acute wound healing process, cell adhesion, proliferation, migration and delineation is mediated by the TGF-β1 dependent signalling pathways including PI3K/AKT and Wnt/β-catenin, forming an interdependence web of signal transduction to achieve effective wound healing [73, 74]. Liang et al. [75], noted that the prosurvival effect of PBM on amyloid β peptide (Aβ) induced apoptosis was attained through the AKT/GSK3β/β-catenin pathway and the inhibition of GSK3β that resulted in the cytosolic accumulation of β-catenin and nuclear translocation. PBM initiates a massive prospective for precise therapy in different types of cutaneous diseases, and the most effective use of PBM necessitates the identification of its mechanisms when light interacts with tissue including the dosage [76, 77]. Wavelength, fluence and frequency of irradiation is critical for cellular mechanisms of PBM in wound healing. Nevertheless, the influence of PBM may differ according to the type of cells, and in this regard, there is still controversy for its use [77–79]. According to Tran et al. [80], it has been reported that LED-light irradiation with wavelengths between 630 and 940 nm has no harmful effects on humans. However, blue LED light with wavelengths of 400–500 nm may be cytotoxic and cause injury to the skin, eyes, and some other human tissues. This danger is popularly known as a ‘‘blue light hazard’’. The clinical demonstration of PBM in different medical disciplines is summarized in Table 1.

**Table 1:** Clinical application of PBM different medical disciplines [81–94]

Medical discipline	Effect
1. Dermatology	• Treatment for skin pigmentation, warts, facial telangiectasias, pyogenic granulomas, hemangiomas, Kaposi’s sarcoma, mucosal diseases and poikiloderma of civatte • Removal of tattoo, acne scars and hair • Stimulate the restoration process in cutaneous lesions pressure ulcers
2. Diabetes or prediabetes	• Antiatherogenic, immunomodulation and antioxidant • Increase myocardial contractility, microcirculation, performance, nerve function • Reduce glycaemia and hemoglobin A1C (HbA1C) • Accelerate angiogenesis in proliferative diabetic retinopathy • Alleviate macular degeneration • Reduces polyneuropathy • Stimulate the restoration process of diabetic wounds
3. Dentistry	• Disinfect and stimulate wound healing • Treatment for oral mucosa malignancies, dentinal hypersensitivity, and herpes simplex • Promotes lymphatic flow and helps to modulate macrophage cell migration from lymphatic vessels • Increases immune response • Reduces swelling and pain • Accelerate the stimulation of fibroblast and osteoblast cells • Rebuilds bones after fractures and orthognathic surgery • Reduces burning mouth syndrome, lichen planus and xerostomia
4. Neural diseases	• Improves recovery from stroke, myocardial infarction, and degenerative and traumatic brain conditions • Reduces brain damage • Treatment for peripheral nerves injuries • Raised levels of neurometabolic energy, and improvement of cerebral hemodynamics and cognitive capabilities

While PBM has been extensively studied, clarifying the fundamental means of its action may advance the understanding of its application, therapeutic benefits, and side effects [95]. In wound healing, PBM at different wavelengths and fluences have been used, resulting in variable data [96]. Variation in PBM protocols in wound management require continued controlled research to get recommendations regarding the desirable parameters [97]. Oliveira et al. [98], noted that a wavelength between 400–800 nm is the most efficient light spectrum in chronic diabetic wounds. The basic mechanism of action and the biological effect of PBM is not clear, arousing interest for research. It is evident that there is a close connection between PBM activated mitochondrial retrograde cellular signalling and molecular processes, including the stimulation and control of cytosolic kinases and downstream cascades for cellular activities required for suitable wound healing [99].

## Conclusions

The management of chronic wounds is of great clinical and public concern, and the causes of delayed and impaired healing in diabetic patients are essential for research and development of novel treatment techniques. The phasic release of GFs/cytokines and the activation of their specific pathways during the wound healing process progresses the healing of acute wounds, and dysregulation of this process contributes to the development of wound chronicity [100–104]. However, the direct interaction of different signalling pathways during wound restoration processes remains uncertain. Therapeutic approaches including single/dual GF, cytokine stimulators and inhibitors, proteinase inhibitors and stem cell therapy are considered for diabetic chronic wounds. There is an interaction between the action of PI3K/AKT and Wnt/β-catenin pathways in cellular proliferation and survival [19]. Treatment methods that enhance the cellular release of cytokines and GFs can augment wound healing and the skin regeneration process. The consideration of the PBM-stimulated biological effects at cellular and molecular levels is key to the advancement of PBMT in the treatment of diabetic wounds [105]. In our effort to understand the molecular effects of PBM on diabetic wounds, we are, in our present work, exploring the relationship and activation of the GSK3β and β-catenin pathways in diabetic wounded cells after PBM in vitro.

## Data Availability

Data sharing is not applicable to this article as no datasets were generated or analysed during the current study.

## Abbreviations

AKT: Protein kinase B
AMPK: AMP-activated protein kinase
APCD1: Adenomatosis polyposis coli down-regulated 1
ATP: Adenosine triphosphates
BAK: BCL-2-antagonist/killer
BAK: Bcl-2 homologous antagonist killer
BCL-2: B cell lymphoma 2
CCO: Cytochrome c oxidase
COX 2: Cyclooxygenase 2
CTNNB1: Catenin beta 1
Dkks: Cerberus, dickkopf proteins
DM: Diabetes mellitus
ECM: Extracellular matrix
EGF: Epidermal growth factor
EGFR: Epidermal growth factor receptor
ERKs: Extracellular signal regulated kinases
ERK-Sp1: ERK-specificity protein 1
FGF: Fibroblast growth factor
FRPs: Frizzled-related proteins
GF: Growth factor
GPC: G-Protein-coupled
GSK3*β*: Glycogen synthase kinase 3 beta
hAMSC: Human amniotic mesenchymal stem cell
IGFBP-4: Insulin-like growth factor binding protein 4
IGFR: IGF receptor
IL: Interleukin
LEDs: Light emitting diodes
LEF: Lymphoid enhancer-binding factor
LLLT: Low level light therapy
MAPK: Mitogen-activated protein kinase
MMPs: Matrix metalloproteinase
mTOR: Mammalian target of rapamycin
mTOR1: Mammalian target of rapamycin complex 1
mTOR2: Mammalian target of rapamycin complex 2
NF-Κb: Nuclear factor kappa-light-chain-enhancer of activated B cell
NO: Nitric oxide
Nrf2: Nuclear factor erythroid 2 related factor 2
PA: Perinatal asphyxia
PBM: Photobiomodulation
PBMT: Photobiomodulation therapy
PDGF: Platelet-derived growth factor
PI3K: Phosphatidylinositol 3 kinase
PI3P_3_: Phosphatidylinositol-3,4,5-trisphosphate
PIP_2_: Phosphatidylinositol 4,5-bisphosphate
PPARs: Peroxisome proliferator-activated receptors
PTEN: Phosphatase and tensin
rhEGF: Recombinant human EGF
ROS: Reactive oxygen species
RTKs: Receptor tyrosine kinases
SH2: Src homology 2
TCF: T cell factor
TGF: Transforming growth factor
TGF‐β: TGF beta
TNF-α: Tumour necrosis factor-alpha
VEGF: Vascular endothelial growth factor
VEGF: Vascular-endothelial growth factor
Waif1: Wnt-activated inhibitory factor 1
WIF-1: Wnt-inhibitory factor 1
Wise/SOST: Sclerostin domain-containing
Wnt: Wingless/integrated
*Α*: Alpha
*Β*: Beta
